# Symmetrical peripheral gangrene caused by septic shock

**DOI:** 10.3109/23320885.2015.1041529

**Published:** 2015-04-28

**Authors:** Keisuke Shimbo, Kazunori Yokota, Junpei Miyamoto, Yukako Okuhara, Mitsuo Ochi

**Affiliations:** 1 ^1^Department of Plastic Surgery, Hiroshima University Hospital, Hiroshima, Japan; 2 ^2^Department of Orthopedic Surgery, Hiroshima University, Graduate School of Biomedical Science, Hiroshima, Japan

**Keywords:** Flap, septic shock, surgical amputation, symmetrical peripheral gangrene

## Abstract

We report three cases of symmetrical peripheral gangrene (SPG) caused by septic shock. Most of sepsis survivors with SPG require amputation of the affected extremities. To preserve the length of the thumb and fingers, we performed surgical amputation and used flaps to cover the amputated peripheral extremities.

## Introduction

Symmetrical peripheral gangrene (SPG) is a rare but severe complication of disseminated intravascular coagulation (DIC) that frequently accompanies sepsis [1,2]. SPG is characterized by symmetric necrosis of the skin and distal extremities, following which two or more distal sites become gangrenous in the absence of large artery occlusion [1,2]. SPG results in high rates of amputation and mortality; in particular, amputation is a serious problem for survivors [1,2]. Multiple distal amputations (e.g., fingers and toes) or more proximal amputations, such as above the knee and elbow, are also well-documented sequelae of this condition.

In the present cases, peripheral ischemia developed within 48–96 h of septic shock onset, and the ischemic changes gradually resulted in gangrene. Surgical amputation was performed for the gangrenous areas on the peripheral extremities, which were covered with a flap following improvements in the patients’ general condition.

It is difficult to prevent the development of gangrene, and amputation cannot be avoided in SPG; however, a correct diagnosis and the appropriate surgical procedure are required to improve patients’ quality of life. These case reports highlight that reconstruction with a flap should be considered to preserve the length of the thumbs and fingers.

## Case reports

### Case 1

**Figure 1.**
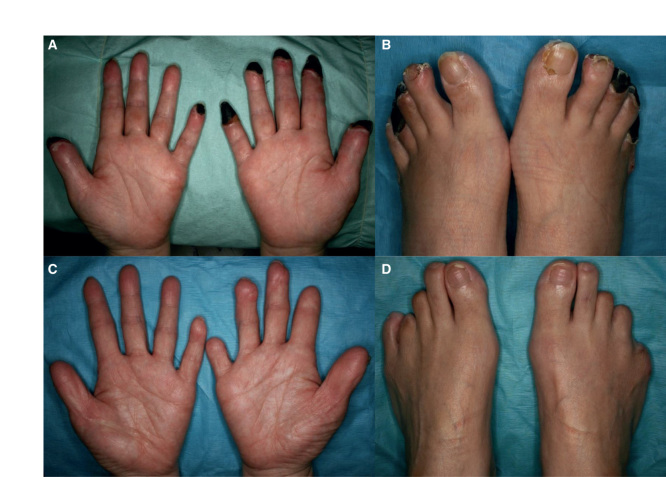
Case 1: (*a*, *b*) Gangrene of several fingers and toes at the initial visit. (*c*, *d*) 1 year postoperatively.

A 58-year-old woman presented to the hospital with fever. Her condition deteriorated to one of septic shock and DIC, and she was admitted to the intensive care unit (ICU). Blood culture results indicated *Pneumococci*. She was treated with a course of antibiotics, vasopressor therapy, a ventilator and continuous hemodiafiltration. Two days after admission, peripheral ischemia appeared on her fingers and toes and gradually resulted in gangrene (Figure 1*a* and *b*). She progressed well clinically over the next few days. One and a half months after admission, skin perfusion pressure (SPP) was measured at eight points (dorsa and palms of both hands and dorsa and plantar of both feet), using SensiLase PAD 3000 (Kaneka Medix Corp. Osaka, Japan). All SPP values were > 50 mmHg. Two months after admission, surgical amputation of 11 gangrenous areas on the peripheral extremities was performed; both thumbs were covered with palmar V-Y advancement flaps [3], and the right four fingers were covered with oblique triangular flaps [4]. There were no postoperative complications. One year after surgery, she was able to carry out activities of daily living (Figure 1*c* and *d*).

### Case 2

**Figure 2.**
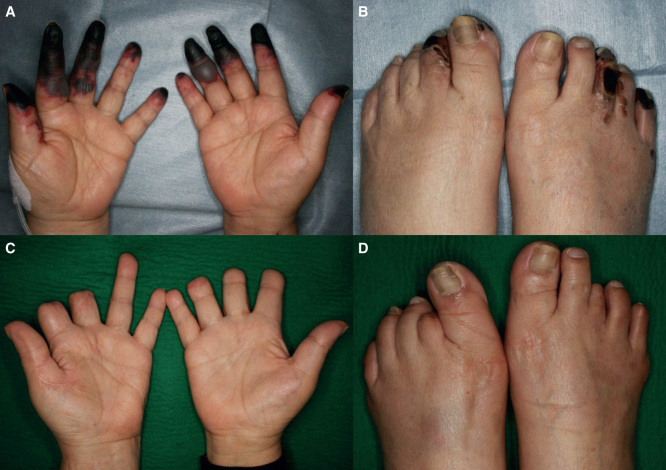
Case 2: (*a*, *b*) Gangrene of several fingers and toes at the initial visit. (*c*, *d*) 1 year postoperatively.

A 63-year-old woman was referred to the hospital with fever. She was diagnosed with septic shock and DIC caused by acute pyelonephritis and admitted to the ICU. Blood culture results indicated *Escherichia coli*. She underwent treatments similar to those for case 1. Four days after admission, peripheral ischemia appeared on her fingers and toes and gradually developed into gangrene (Figure 2*a* and *b*). She progressed well clinically over the next few days. One month after admission, SPP values > 50 mmHg were detected on the dorsa and palms of both hands and the dorsa and plantar of both feet. Surgical amputation of 10 gangrenous areas on the peripheral extremities was performed 36 days after admission, and the left thumb was covered with a palmar V-Y advancement flap. There were no postoperative complications. One year after surgery, she was able to carry out activities of daily living (Figure 2*c* and *d*).

### Case 3

**Figure 3.**
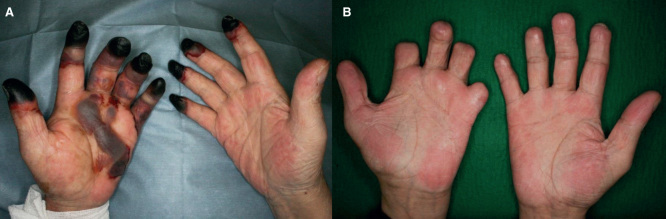
Case 3: (*a*) Gangrene of several fingers at the initial visit. (*b*) 1 year postoperatively.

A 59-year-old man was referred to the hospital with fever and swelling of the right thigh. He was admitted to the ICU with a diagnosis of gas gangrene. He progressed to septic shock and DIC. Blood culture results showed negative but pus culture results indicated *Corynebacterium bacteroides fragilis*. Four days after admission, a hip disarticulation was performed because of worsening of the gas gangrene; at the same time, peripheral ischemia appeared on the fingers of both hands (Figure 3*a*). He then underwent treatments similar to those for case 1 and recovered slowly. One and a half months after admission, SPP values > 60 mmHg were measured on the dorsa and palms of both hands. Two months after admission, surgical amputation of nine gangrenous areas on the peripheral extremities was performed; the left thumb was covered with a palmar V-Y advancement flap, and the right four fingers were covered with oblique triangular flaps. One year after surgery, despite the loss of his right lower limb, he was able to carry out activities of daily living with his hands (Figure 3*b*).

## Discussion

SPG is uncommon condition caused by DIC, hemodynamic compromise, and sepsis. DIC is involved in up to 85% of cases of SPG [2]. SPG is usually observed as a complication of various infective diseases. *Meningococci, Pneumococci, E. coli, Pseudomonas* and *Klebsiella* have been identified as causative factors [5]. In our three cases, the causative microorganisms and field disease were different, but they followed the same course to SPG. In SPG, coagulation is disrupted as a result of DIC. DIC results in intravascular thrombosis and infarction of the skin and distal extremities. The resulting state of low blood flow results in thrombotic occlusion of the microcirculation of the affected extremities. The use of vasopressors is simultaneously involved in spasm of the vessels and aggravation of the microcirculation [6]. Pathologic examination of amputated specimens often reveals thrombi concentrated in the small vessels and not the large vessel [7].

The treatment priority is usually the underlying condition and DIC; therefore, SPG is typically not treated immediately. In certain situations, this devastating complication may not be avoidable; therefore, thorough diagnostic examinations and appropriate management are required for SPG. Large vessel occlusion or vasculitis is often not present with SPG; however, it is important to diagnose the presence of compromised blood flow to the extremities. In such a condition, minimally invasive examinations such as SPP may be the best diagnostic tools. Debridement can reportedly be conducted at an amputation level where SPP is > 35 mmHg [8]. In our cases, because SPP was considerably greater than 35 mmHg, we determined that the SPG could be cured with surgery.

SPG should be suspected with presenting coldness, pallor, cyanosis or pain in the distal extremity in the initial phases. The affected extremities may progress to erythema or purpural lesions that subsequently develop into gangrene [7]. It is important to distinguish SPG from other forms of gangrene (e.g., blue toe syndrome) and to identify the associated important skin lesions (e.g., purpura fulminans [PF]) by the extracutaneous clinical features and pathogenesis. Blue toe syndrome is characterized by tissue ischemia secondary to cholesterol crystal or atherothrombotic embolization leading to occlusion of small vessels [9]. Unlike SPG, blue toe syndrome is most often caused by embolization such as invasive vascular procedures, anticoagulation or thrombolytic therapy. SPG appears very similar to PF in the pathophysiology. SPG is often described as synonymous to acute infectious PF [10].

There is currently no specific prevention and treatment for SPG. Approximately, more than half of the patients that recover from SPG require amputation of the affected limb [2]. Amputation is rarely urgently required because ischemia of the extremities is potentially reversible, and secondary infection of the necrotic tissue is uncommon. Amputation should be considered only after the patient’s condition improves and the gangrenous areas become demarcated [11]. Too much viable tissue is lost by amputation. Therefore, an appropriate surgical procedure, including the choice of flap, should be selected. In our cases, we performed surgical amputation for a total of 30 gangrenous areas and covered the maintained joint function of thumbs and fingers with flaps. Many skin flaps are currently available, and it can be difficult to choose a flap from the many available techniques. First, we selected an operative method to reconstruct the thumb and fingers based on the aim of preserving their length as much as possible. Second, we chosed a flap that could be completed in a short time because the patients had only recently recovered from sepsis and had multiple gangrenous areas. Palmar V-Y advancement flaps were transferred in four thumbs, and oblique triangular flaps were used in eight fingers. There were no postoperative complications in any of the flaps, and three patients were able to carry out activities of daily living with their hands. Previous reports have indicated that free flaps can be particularly effective for salvaging limbs [12]. Salvage of the length of the extremities is extremely important for maintaining the patients’ quality of life.

Further studies are required to determine the etiology, prevention and appropriate treatment of SPG. It is crucial that the majority of doctors are able to recognize SPG and select the appropriate treatment for their patients. We consider reconstruction with flaps to be one of the most effective surgical treatment options for SPG.

***Declaration of interest:*** The authors report no conflicts of interest. The authors alone are responsible for the content and writing of the paper.
